# Using preimplantation genetic testing for monogenic disease for preventing citrullinemia type 1 transmission

**DOI:** 10.3389/fgene.2024.1389461

**Published:** 2024-08-08

**Authors:** Zubo Wu, Tao Liang, Yi Liu, Xiaofang Ding, Defeng Shu

**Affiliations:** ^1^ Department of Pediatrics, Union Hospital, Tongji Medical College, Huazhong University of Science and Technology, Wuhan, China; ^2^ Department of Clinical Laboratory, Union Hospital, Tongji Medical College, Huazhong University of Science and Technology, Wuhan, China; ^3^ Department of Obstetrics and Gynecology, Union Hospital, Tongji Medical College, Huazhong University of Science and Technology, Wuhan, China

**Keywords:** preimplantation genetic test, citrullinemia type 1, ASS1, allelic dropout, haplotype

## Abstract

**Aim:**

The aim of this study is to investigate if Preimplantation Genetic Testing (PGT) can effectively identify unreported variants according to American College of Medical Genetics and Genomics (ACMG)to prevent citrullinemia type 1 affection.

**Design:**

This study involves a detailed case analysis of a family with history of citrullinemia type 1, focusing on the use of PGT for monogenic diseases (PGT-M). The genetic variants were identified using ACMG guidelines, and PGT was employed to prevent the inheritance of these variants. The study included haplotype analysis and Sanger sequencing to confirm the results.

**Results:**

The study identified previously unreported variations in the ASS1 gene causing citrullinemia type 1. PGT successfully prevented the transmission of these variants, resulting in the birth of a healthy fetus. However, challenges such as allele dropout (ADO) and gene recombination were encountered during haplotype analysis, which could potentially defeat the diagnosis. The study demonstrated that combining haplotype analysis with Sanger sequencing can enhance the accuracy of PGT.

**Conclusion:**

Preimplantation Genetic Testing (PGT) targeting likely pathogenic and pathogenic variants in the ASS1 gene, as rated by ACMG, allows the birth of healthy infants free from citrullinemia type 1. Additionally, the establishment of single haplotypes and Sanger sequencing can reduce the misdiagnosis rate caused by allele dropout (ADO) and genetic recombination.

## Introduction

Inherited metabolic diseases are rare in the general population but can cause significant harm to affected families and society due to their often untreatable nature (Ferreira and van Karnebeek, 2019). Citrullinemia type 1, with an incidence rate of about 1/22,000, is characterized primarily by high blood ammonia levels (Posset et al., 2020). Symptoms range from mild to severe and can include increased intracranial pressure, heightened neuromuscular tension, spasticity, seizures, loss of consciousness, and even death due to elevated blood ammonia and other toxic substances (Kölker et al., 2015). Citrullinemia type 1 can lead to intellectual disability or death despite the availability of effective therapies (Häberle et al., 2019). Preimplantation genetic testing (PGT) offers hope for affected families by preventing intergenerational inheritance (Cho et al., 2014). High-risk families can use PGT to prevent genetic diseases in offspring, making gene diagnosis crucial throughout the process.

Since the American College of Medical Genetics and Genomics (ACMG) issued standards for classifying unreported gene variants in 2015, a unified standard has been established (Richards et al., 2015). However, the efficacy of these standards in successfully blocking the transmission of inherited metabolic diseases requires further clinical support (Chen, 2023). Mutations in ASS1 can cause citrullinemia type 1 (Xiong and Chen, 2022), and functional tests showed that mutation in ASS1 affects its expression (Liu et al., 2023). In this research, we present a family case of citrullinemia type 1 involving previously unreported mutation sites in ASS1, classified as pathogenic or likely pathogenic according to ACMG guidelines. Preimplantation genetic testing for monogenic disease (PGT-M) was performed, followed by monitoring related conditions until birth.

## Materials and methods

### Ethical approval

This study involving human participants was reviewed and approved by Union Hospital, Wuhan, China. The patients/participants all provided written informed consent to participate in this study. Written informed consent was obtained from the authors and participating patients for the publication of any potentially identifiable images or data included in this article.

### Clinical data

The female partner was 32 years old, with a height of 158 cm, body mass index (BMI) of 19.6, and Anti-müllerian hormone (AMH) level of 2.6 ng/mL. Both partners denied having a consanguineous marriage and reported no family history of congenital diseases. The couple had two natural births, with both the first and second child diagnosed with citrullinemia type 1. Probands in this family were dead because of the citrullinemia type 1, making pedigree analysis impossible for them.

### Genetic diagnosis of the proband

Whole exome sequencing was done for the second child of the family who was affected of citrullinemia type 1. The results revealed mutations ASS1:c.847G>A (originating from the male partner), rated as P (pathogenic): Evidence: [PM3_Very strong + PM1+PM2_Supporting + PP3]; and c.1127 + 1G>T (originating from the female partner), rated as LP (likely pathogenic) with Evidence: PVS1+PM2_Supporting. This variation was not reported in citrullinemia type 1 patients according to American College of Medical Genetics and Genomics (ACMG).

### Pre-testing and pedigree analysis

A total of 5 mL of peripheral blood was collected from both male and female partners and their respective parents (six individuals in total) for genomic DNA (gDNA) extraction. Oral mucosal cells were also collected from these six individuals for single-cell whole genome sequencing using multiple annealing and looping-based amplification cycles (MALBAC). Sanger sequencing was conducted to verify the *ASS1* gene mutation sites c.1127 + 1G>T and c.847G>A. It confirmed that the male partner carried the c.847G>A, p. E283K missense mutation, inherited from his father, and the female partner carried c.1127 + 1G>T, an RNA splicing mutation inherited from her father. Additionally, single nucleotide polymorphism (SNP) genotyping was conducted on the gDNA samples from the family within a 2-Mb range upstream and downstream of the *ASS1* gene using the Illumina iScan Reader and the Infinium Asian Screening Array-24 v1.0 BeadChip to identify haplotypes associated with the disease-causing mutations and to establish a foundation for subsequent embryonic SNP linkage analysis.

### PGT-M treatment

PGT-M treatment was initiated after successful pre-testing using the PPOS protocol to ovarian stimulation, 7 oocytes were retrieved, Intracytoplasmic sperm injection (ICSI) was utilized for fertilization,2 blastocyst were biopsied and vitrified, the second cycle was initiated with GnRH antagonist protocol because of there is no embryos can be used from the PGT results. A total of 16 oocytes were retrieved, which included 13 MII oocytes. (ICSI) was utilized for fertilization, and the embryos were cultured up to day 5. Trophectoderm biopsy and genetic testing were performed on six morphologically useable blastocysts, which were then vitrified.

### Single-cell whole genome amplification and sequencing

Trophectoderm biopsies were carried out, and whole genome amplification (WGA) was performed using a universal sample processing kit, ChromSwiftTM (XK-028, Yikon Genomics) for gene sequencing according to the manufacturer’s instructions. WGA products were fragmented for library construction and sequenced on the Illumina Nextseq 550 platform to analyze the ploidy of each embryo. SNP haplotype analysis of the WGA products and first-generation sequencing of mutation loci were also conducted to determine the genotype of the embryos. Finally, based on the results of SNP linkage analysis of the mutated gene of the embryo, verifications of the point mutations, and the aneuploidy testing results, embryos that did not carry disease-causing mutations and had normal ploidy were selected for clinical transplantation.

### Analysis of copy number variations (CNVs)

After the removal of duplicates from the original reads, they were mapped to the genome in 1 Mb units (bins) across the entire genome and standardized by the GC content (the proportion of guanine and cytosine) and a reference data set. When the copy number of each bin increased from 2 to 3, the number of reads increased by 50%, and when it decreased from 2 to 1, the number of reads decreased by 50%. The circular binary segmentation algorithm (CBS) was used to report embryonic CNVs of ≥4 Mb, and the R program was used to visualize the CNVs of each bin of the 24 chromosomes.

For SNP site analysis, data from high-throughput sequencing were mapped to the human reference genome (hg19). Further analysis of the family members was conducted, along with the detection of whole genome SNP sites in the test samples. SNP sites within 1–2 Mb upstream and downstream of the pathogenic gene were selected. Finally, a report was presented based on thorough evaluation of the SNP linkage analysis results of the mutated gene in the embryos, confirmation of the point mutations, and the aneuploidy testing results.

### Embryo transfer and follow-ups

On the second day of the menstrual cycle of the female partner, preparations for thawing and transfer were made following an artificial cycle protocol. Endometrial preparation was performed using progesterone injections, and embryo transfer was conducted on the fifth day of progesterone administration. Routine luteal support was maintained through 12 weeks of gestation, followed by amniocentesis at 18 weeks to verify the fetal genotype.

## Results

### Results of PGT-M and follow-ups

**FIGURE 1:**
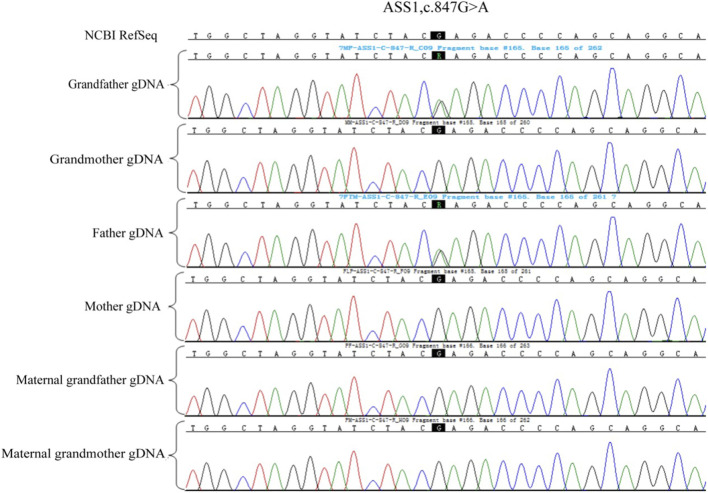
Validation of ASS1: c.847G>A mutation in the family.

**FIGURE 2:**
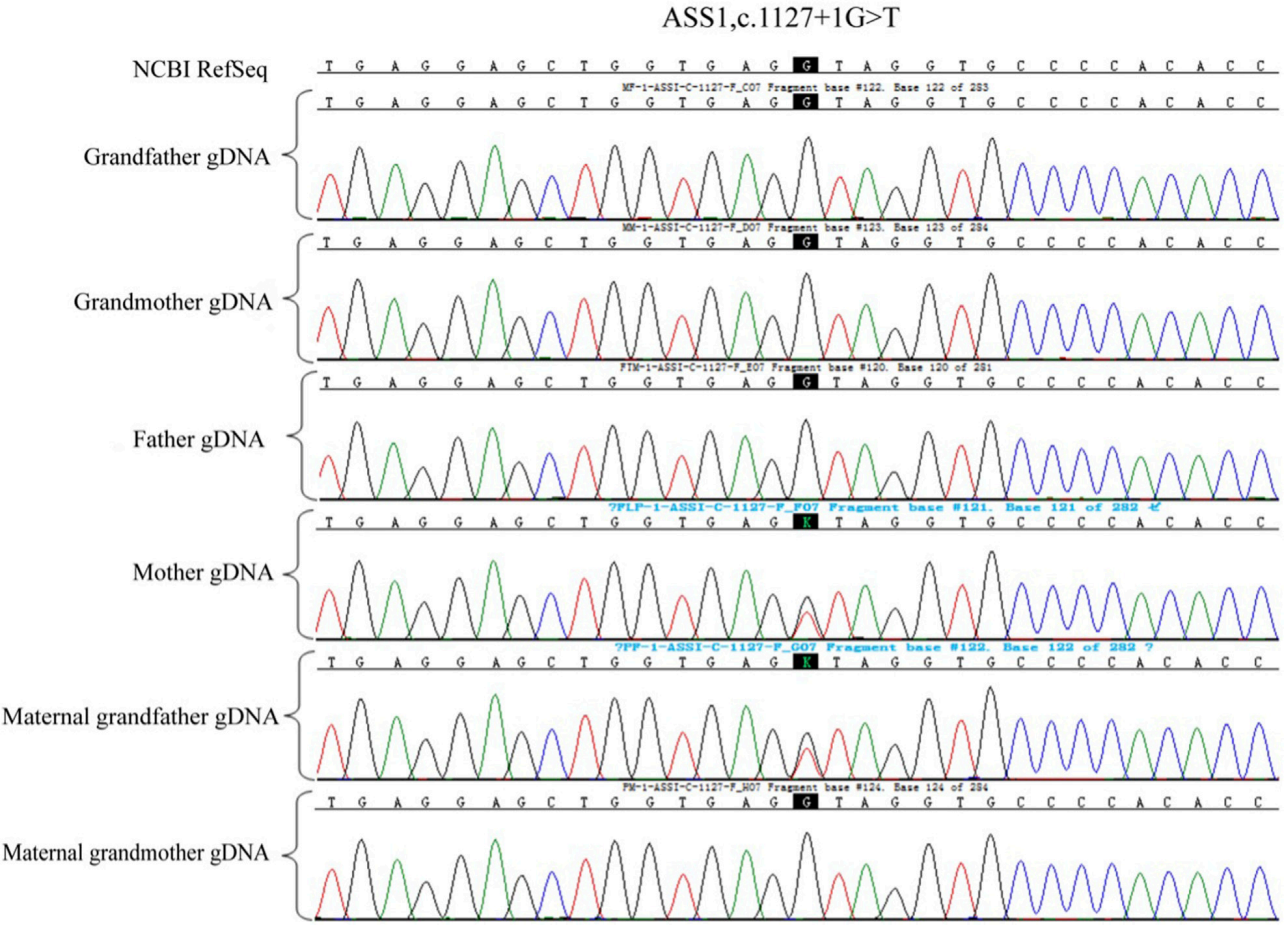
Validation of ASS1: c.1127 + 1G>T mutation in the family.

**FIGURE 3:**
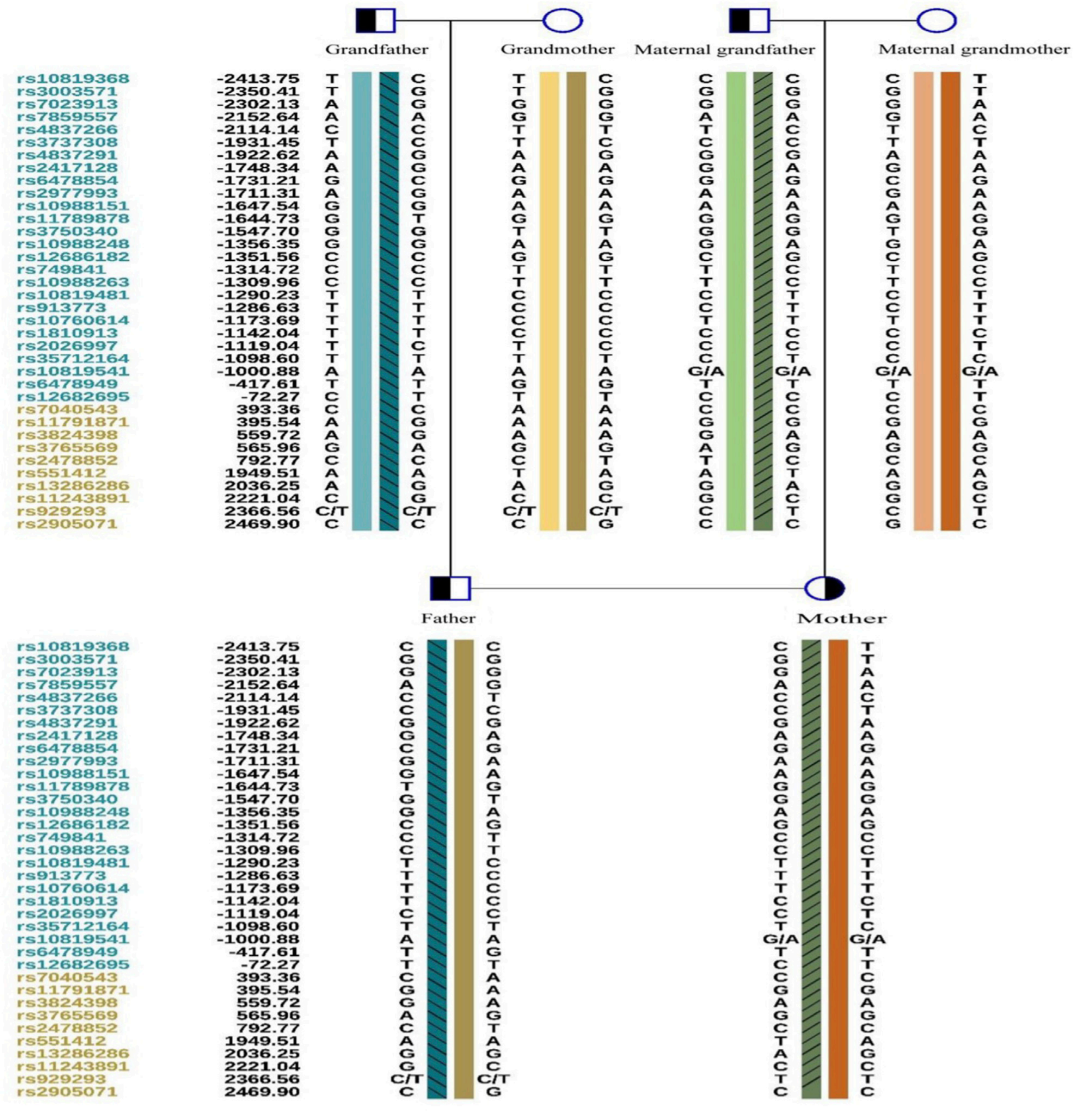
Haplotypes by SNP linkage of the two mutations in this Family.

Owing to the unavailability of a proband sample and DNA, verification of the genotypes of the family and haplotype establishment were performed using samples from the parents of both partners. Figures 1–3 show the Sanger sequencing results and haplotype results by SNP in the family.

### Embryo testing results

**FIGURE 4:**
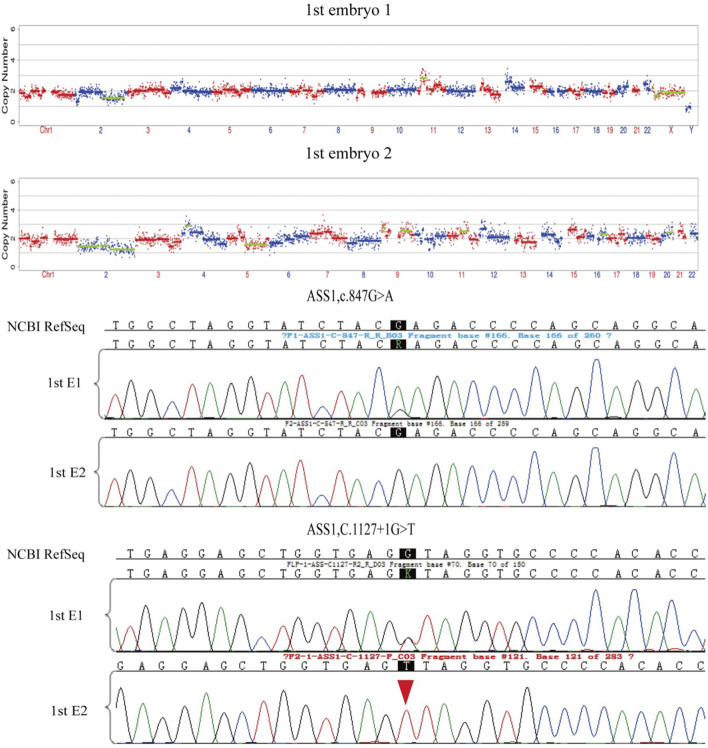
The mutations test and CNV test results of the two embryos of the first cycle of PGT. Red arrows show the ADO from the haplotype analysis in Figure 5. All the two embryos are aneuploid.

**FIGURE 5:**
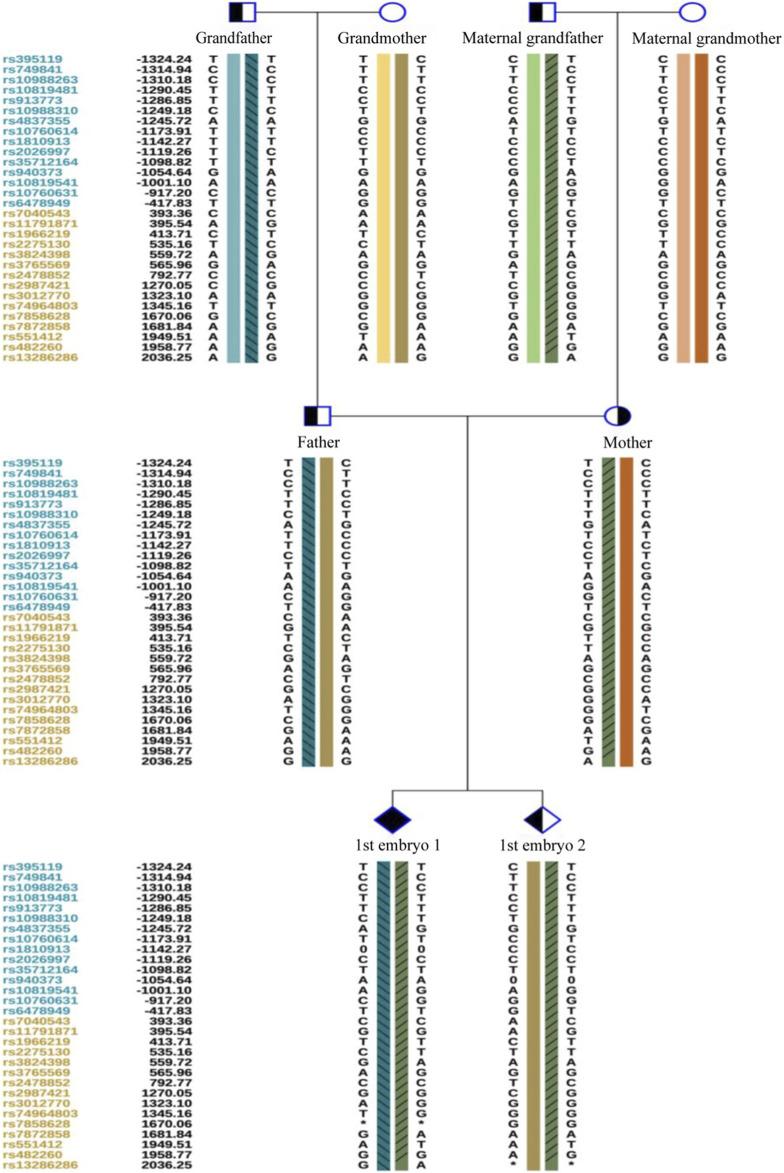
The haplotype of SNP analysis of the two embryos in the first cycle of PGT. Embryo 1 carried the two mutations and embryo 2 only carried the maternal mutation.

**FIGURE 6:**
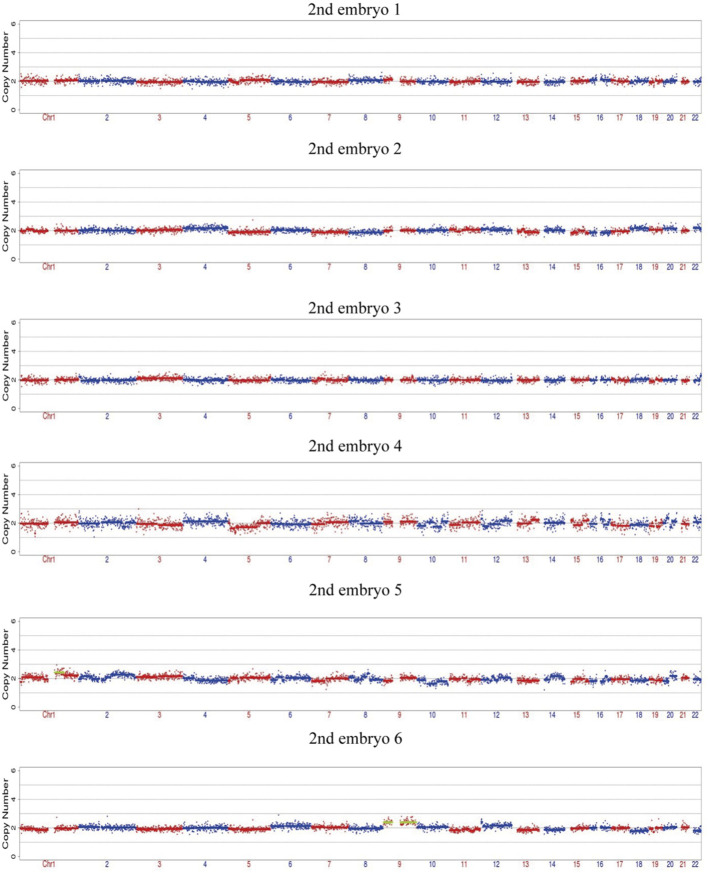
The CNV test of the 6 embryos of the second cycle of PGT, four embryos are euploid, two embryos are mosaic.

**FIGURE 7:**
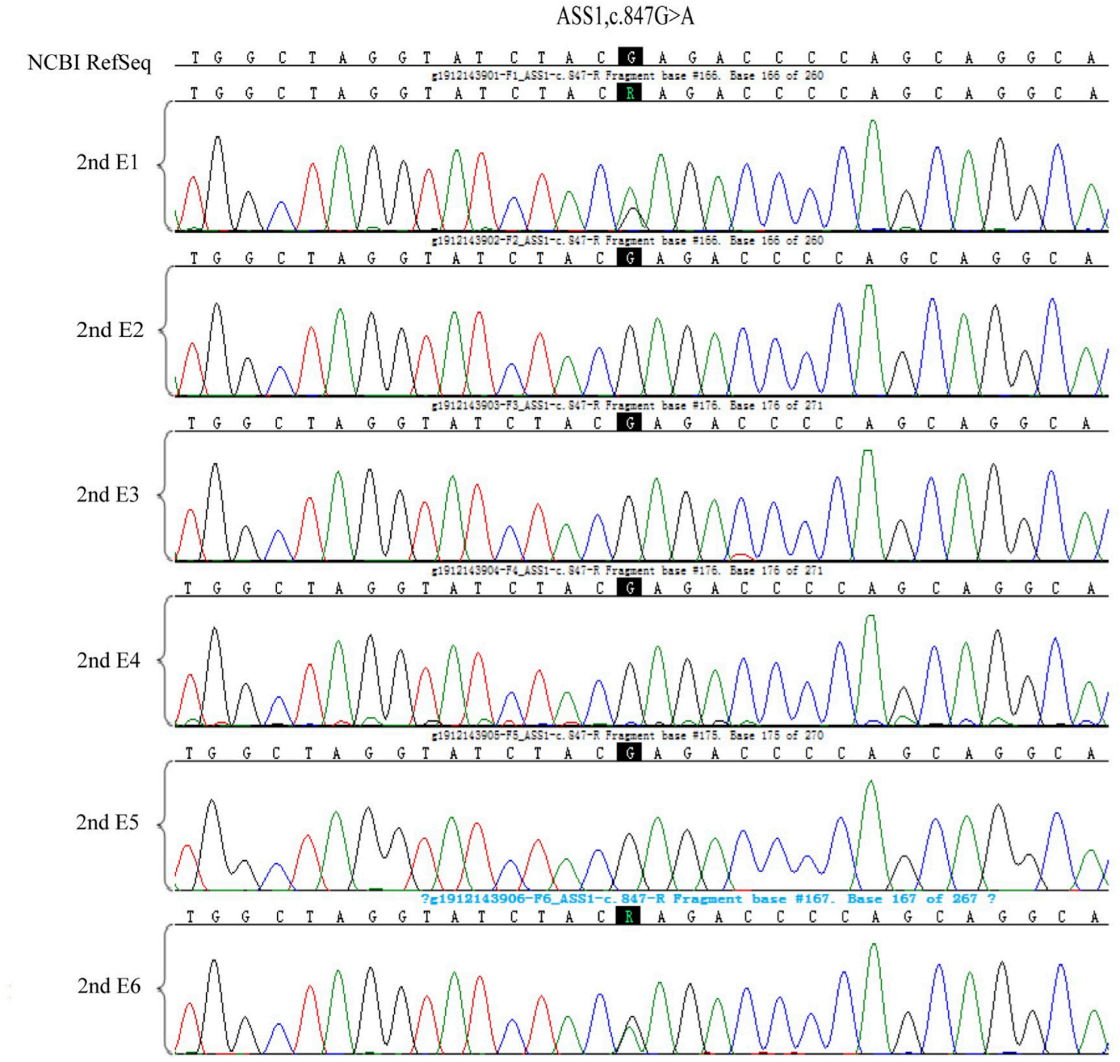
The ASS1:c.847G>A test results in the 6 embryos of the second cycle of PGT-M.

**FIGURE 8:**
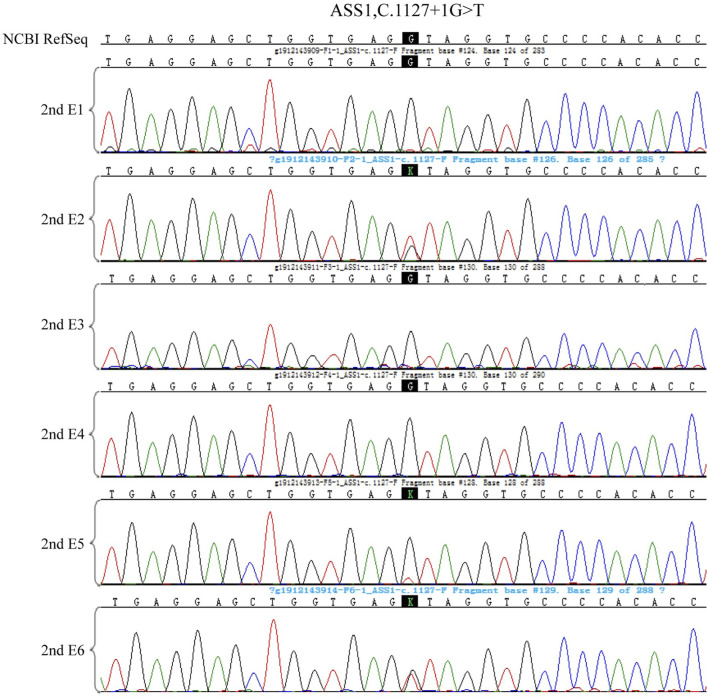
The ASS1: c.1127 + 1G>T test results in the 6 embryos of the second cycle of PGT-M.

**FIGURE 9:**
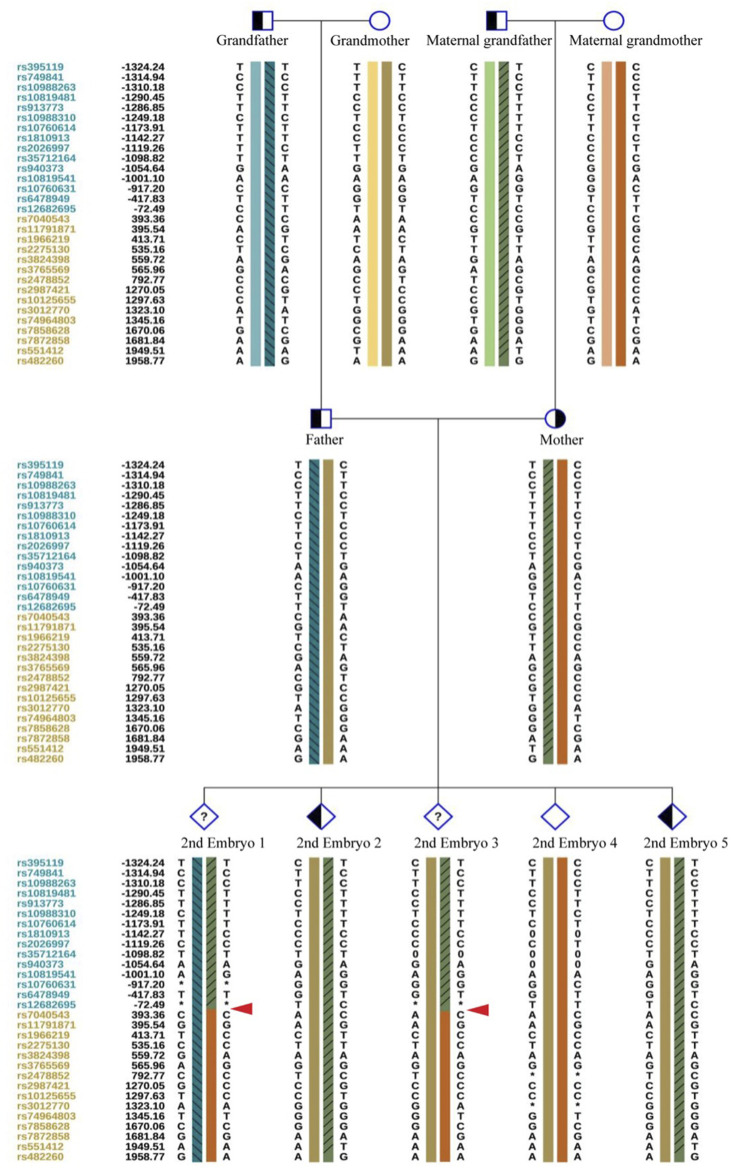
The haplotype of SNP analysis of the six embryos in the second cycle of PGT. Red arrows show the recombination of the gene sequence. Haplotype of SNP analysis was defeated in embryo 6 because of the mosaic state of 9 chromosome, the ASS1 gene is locate on 9q34.11.

After the successful establishment of the haplotypes, the couple’s first *in vitro* retrieval yielded two blastocysts, both with chromosome aneuploidies, and thus no blastocyst was suitable for transfer. Later, six blastocysts were obtained through another round of *in vitro* fertilization. Among these, one blastocyst, free of mutations from either partner and with a euploid chromosomal constitution, was thawed and transferred, leading to a successful pregnancy. Figures 4–9 shows the embryos test results of the two cycles of PGT including CNV, mutation test and haplotype analysis.

### Follow-up results of amniocentesis

**FIGURE 10:**
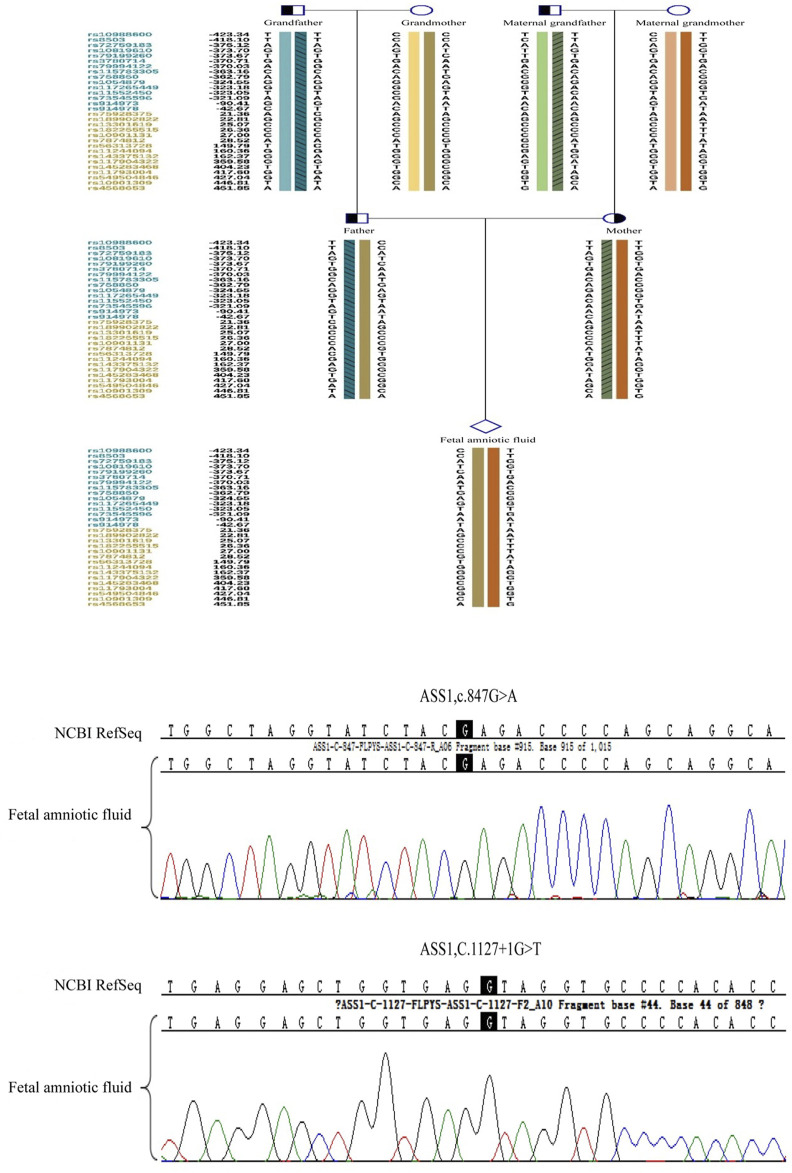
The prenatal testing results of the fetal amniotic fluid by the sanger sequence and haplotype analysis of SNP.

Amniocentesis was performed 18 weeks into the successful pregnancy. Verification of the point mutations and aneuploidy testing were repeated. After ruling out maternal genomic DNA contamination, the results confirmed that the fetus did not carry the genetic mutations of the parents. The child was born healthy and currently shows no related phenotypes. Figure 10 shows the haplotype and mutation test results.

## Discussion

Citrullinemia type 1 symptoms vary widely, and without early diagnosis and treatment, the prognosis for children with severe symptoms is very poor (Häberle et al., 2019). Therefore, PGT and prenatal diagnosis are critically important. Our data provide further clinical evidence for preventing citrullinemia type 1 in a family, including observing allele dropout (ADO) during testing and gene recombination during generational transmission.

Current guidelines and consensus recommendations primarily address medical interventions for variants classified as pathogenic or likely pathogenic. However, the potential for diagnostic errors in identifying such variants and the risk of misdiagnosis in PGT remain (Chen, 2023). In this case, the ASS1.847G>A variant, originating from the male partner, is a pathogenic missense mutation. Previous reports suggest that this mutation may affect the folding of specific protein domains, although no affected patients have been documented. The evidence for this variant is classified as PM3_Very strong + PM1+PM2_Supporting + PP3 according to ACMG guidelines (Gao et al., 2003).

The c.1127 + 1G>T variant, originating from the female partner, is classified as likely pathogenic. It is a previously unreported alternative splicing variant categorized as potentially causative. The evidence type for this variant is PVS1+PM2_Supporting according to ACMG guidelines (Engel, Höhne, and Häberle 209). This splicing variant occurs at the donor site with a SpliceAI score of 0.99. Given the ACMG rating of likely pathogenic and the clinical diagnosis of the related disease corresponding to this gene variant (PP3), this variant could be considered pathogenic. As the variant is classified as pathogenic, we did not further perform functional studies to assess protein expression abnormalities caused by this splicing site, such as exon skipping, intron inclusion, or cryptic splice site usage. However, these functional validations are crucial for understanding the disease’s pathogenesis.

Determining the embryonic genotype based on haplotypes is a commonly used technique in genetic diagnosis of embryos (Harton et al., 2011). Establishing haplotypes through family members clarifies the haplotype where the genetic variant is located, effectively reducing the risk associated with allele dropout during embryonic genetic testing. However, diagnostic errors due to genetic recombination remain possible, underscoring the importance of prenatal diagnosis (Wilton et al., 2009). This case includes a detailed description of citrullinemia type 1 occurrence, diagnosis, tracing, prevention, verification, and follow-up in a family. We observed ADO and recombination, which can complicate embryonic diagnosis, but haplotype analysis combined with Sanger sequencing can increase PGT success rates.

ADO is a phenomenon observed during PCR, potentially leading to genetic diagnostic errors (Blais et al., 2015), ADO occurrence may be related to high CG content (Wenzel et al., 2009), and the amount of PCR amplification template and analysis methods (Hedell et al., 2015), Despite mitigating high-risk factors, ADO cannot be completely avoided (Blais et al., 2015). This is more likely to occur in embryonic testing, where linkage analysis can reduce misdiagnosis rates caused by ADO (Rechitsky et al., 1998). However, genetic recombination during haplotype determination can affect diagnosis. In this study, we observed two recombination events in the tested region over three generations, possibly due to this region being a recombination hotspot. Our data support varying recombination regions across different embryos. The necessity of simultaneous CNV testing during PGT-M is debated (Yang et al., 2022), but our data indicate that concurrent CNV testing can effectively reduce embryo aneuploidy rates.

Amniocentesis verification confirmed the fetal genotype, underscoring the importance of prenatal diagnosis in accurately assessing fetal genetic risks. Unlike PCR, prenatal diagnosis does not carry ADO-related risks and involves cells from multiple embryonic layers, offering more accurate evaluations than trophoblast cell sampling during the embryonic stage (Kahraman et al., 2020; Greco et al., 2023).

Although our research suggests that clinical intervention through PGT methods could prevent the birth of children with citrullinemia type 1, our study has several limitations. Firstly, expanding the sample size of citrullinemia type 1 patients is necessary to further validate the effectiveness of PGT methods in preventing the transmission of ASS1 gene mutations. Establishing the relationship between genetic variants and phenotypic outcomes is crucial for PGT. While the ACMG guidelines offer valuable references, many variants lack clear causal relationships with phenotypes, emphasizing the necessity for further research and clinical data. Furthermore, our study did not include functional studies on the two variants identified, which are essential for understanding changes in protein function and the underlying pathogenic mechanisms. Additionally, there is a need for further research on the accuracy and detection methods of PGT. Based on the above problems, future research will involve conducting scientific experiments to verify our findings and enhance the understanding of PGT’s potential in managing citrullinemia type 1 Engel et al., 2009.

## Conclusion

Preimplantation Genetic Testing (PGT) targeting likely pathogenic and pathogenic variants in the ASS1 gene, as rated by ACMG, allows the birth of healthy infants free from citrullinemia type 1. Additionally, the establishment of single haplotypes and Sanger sequencing can reduce the misdiagnosis rate caused by allele dropout (ADO) and genetic recombination.

## Data Availability

The original contributions presented in the study are publicly available. This data can be found here: http://www.ncbi.nlm.nih.gov/bioproject/1142457.
